# Risk, Unexpected Uncertainty, and Estimation Uncertainty: Bayesian Learning in Unstable Settings

**DOI:** 10.1371/journal.pcbi.1001048

**Published:** 2011-01-20

**Authors:** Elise Payzan-LeNestour, Peter Bossaerts

**Affiliations:** 1 University of New South Wales, Sydney, Australia; 2 California Institute of Technology, Pasadena, California, United States of America; 3 Swiss Finance Institute at EPFL, Lausanne, Switzerland; John Radcliffe Hospital, United Kingdom

## Abstract

Recently, evidence has emerged that humans approach learning using Bayesian updating rather than (model-free) reinforcement algorithms in a six-arm restless bandit problem. Here, we investigate what this implies for human appreciation of uncertainty. In our task, a Bayesian learner distinguishes three equally salient levels of uncertainty. First, the Bayesian perceives irreducible uncertainty or risk: even knowing the payoff probabilities of a given arm, the outcome remains uncertain. Second, there is (parameter) estimation uncertainty or ambiguity: payoff probabilities are unknown and need to be estimated. Third, the outcome probabilities of the arms change: the sudden jumps are referred to as unexpected uncertainty. We document how the three levels of uncertainty evolved during the course of our experiment and how it affected the learning rate. We then zoom in on estimation uncertainty, which has been suggested to be a driving force in exploration, in spite of evidence of widespread aversion to ambiguity. Our data corroborate the latter. We discuss neural evidence that foreshadowed the ability of humans to distinguish between the three levels of uncertainty. Finally, we investigate the boundaries of human capacity to implement Bayesian learning. We repeat the experiment with different instructions, reflecting varying levels of structural uncertainty. Under this fourth notion of uncertainty, choices were no better explained by Bayesian updating than by (model-free) reinforcement learning. Exit questionnaires revealed that participants remained unaware of the presence of unexpected uncertainty and failed to acquire the right model with which to implement Bayesian updating.

## Author Summary

The ability of humans to learn changing reward contingencies implies that they perceive, at a minimum, three levels of uncertainty: risk, which reflects imperfect foresight even after everything is learned; (parameter) estimation uncertainty, i.e., uncertainty about outcome probabilities; and unexpected uncertainty, or sudden changes in the probabilities. We describe how these levels of uncertainty evolve in a natural sampling task in which human choices reliably reflect optimal (Bayesian) learning, and how their evolution changes the learning rate. We then zoom in on estimation uncertainty. The ability to sense estimation uncertainty (also known as ambiguity) is a virtue because, besides allowing one to learn optimally, it may guide more effective exploration; but aversion to estimation uncertainty may be maladaptive. Here, we show that participant choices reflected aversion to estimation uncertainty. We discuss how past imaging studies foreshadowed the ability of humans to distinguish the different notions of uncertainty. Also, we document that the ability of participants to do such distinction relies on sufficient revelation of the payoff-generating model. When we induced structural uncertainty, participants did not gain awareness of the jumps in our task, and fell back to model-free reinforcement learning.

## Introduction

In an environment where reward targets and loss sources are stochastic, and subject to sudden, discrete changes, the key problem humans face is learning. At a minimum, they need to be able to assess *estimation uncertainty* [1]–[4], i.e., the extent to which learning still has to be completed. High levels of estimation uncertainty call for more learning, while low levels of estimation uncertainty would suggest slower learning.

To correctly gauge estimation uncertainty, two additional statistical properties of the environment ought to be evaluated: *risk*, or how much irreducible uncertainty would be left even after the best of learning; and *unexpected uncertainty*, or how likely it is that the environment suddenly changes [5]. The notion of risk captures the idea that, to a certain extent, forecast errors are expected, and therefore should not affect learning. Under unexpected uncertainty, these same forecast errors are indications that learning may have to be re-started because outcome contingencies have changed discretely.

With Bayesian learning, the three notions of uncertainty are tracked explicitly. This is because Bayesians form a model of the environment that delineates the boundaries of risk, estimation uncertainty and unexpected uncertainty. The delineation is crucial: estimation uncertainty tells Bayesians how much still needs to be learned, while unexpected uncertainty leads them to forget part of what they learned in the past.

This contrasts with model-free reinforcement learning. There, uncertainty is monolithic: it is the expected magnitude of the prediction error [6]. Under reinforcement learning, only the value of a chosen option is updated, on the basis of the reward (or loss) prediction error, i.e., the difference between the received and the anticipated reward (or loss) [7]. No attempt is made to disentangle the different sources of the prediction error. Usually, the learning rate is kept constant. If not, as in the Pearce-Hall algorithm [8], adjustment is based on the *total* size of the prediction error.

Recently, evidence has emerged that, in environments where risk, estimation uncertainty and unexpected uncertainty all vary simultaneously, humans choose as if they were Bayesians [9]. Formally, the experiment that generated this evidence involved a six-arm restless bandit problem. Participants were asked to choose among six options with different risk profiles and differing frequencies of changes in reward (and loss) probabilities. Assuming softmax exploration [10], the Bayesian updating model was shown to provide a significantly improved fit over standard reinforcement learning as well as the Pearce-Hall extension.

To discover that humans are Bayesians implies that they must have tracked the three levels of uncertainty. Here, we discuss how the levels differentially affected the Bayesian learning rate in our restless bandit task, and how participants could have distinguished between them.

Neural implementation of Bayesian learning would require separate encoding of the three levels of uncertainty. Recent human imaging studies appear to be consistent with this view. The evidence has only been suggestive, however, as no imaging study to date involved independent control of risk, estimation uncertainty and unexpected uncertainty.

Indeed, to our knowledge, ours is the first comprehensive study of risk, estimation uncertainty, and unexpected uncertainty. Many studies have focused on risk [11]–[13]. Estimation uncertainty has been investigated widely in the economics literature, where it is referred to as ambiguity [14], and a few imaging studies have explored its neurobiological basis [3], [15], [16]. Unexpected uncertainty has only rarely been considered [4], [5]. [4] is closest to our study in that it was the first to document that humans correctly adjust their learning rates to changes in the average level of unexpected uncertainty (referred to in [4] as *volatility*).

The task in [4] involved a bandit with only two arms, however. For our purposes, this entails a number of disadvantages. First, it is impossible to independently track the three levels of uncertainty with only two arms; at a minimum, six arms are needed, and this is what is implemented in the experiment here. As a matter of fact, in [4], risk was decreased along with unexpected uncertainty, introducing a confound that masked the full effect of unexpected uncertainty on the learning rate. Second, the two arms in [4] have perfectly negatively correlated reward probabilities, and as such, the task is one of reversal learning [17]. This means that outcomes for one arm are fully informative for the other one. Consequently, exploration is of no consequence.

This is important because, here, we are interested in re-visiting the data in [9] and investigate exploration. One of the notions of uncertainty, namely, estimation uncertainty, is not only an important determinant of the learning rate. It has been conjectured to be a key driving force behind exploration. Specifically, some have proposed that an “exploration bonus” be added to the value of an option, and that this exploration bonus be increased with the need to learn, i.e., with estimation uncertainty [1], [10], [18].

In our six-arm restless bandit problem, estimation uncertainty varied substantially over time and across arms, thus providing power to detect the presence of an exploration bonus in valuation, and hence, an effect of estimation uncertainty on exploration. Before our study, behavioral evidence in favor of an exploration bonus had been weak: [10] showed that human exploration can be modeled using softmax, but found no reliable evidence of an exploration bonus. But in their (four-armed) bandit problem, estimation uncertainty varied little across bandits, unlike in ours.

Firing of dopaminergic neurons in response to novel, uncertain stimuli has been interpreted as signaling exploration value [18]; yet, it can be questioned whether estimation uncertainty ought to enter valuation through a bonus. Findings in economics, starting with [19], would make one believe otherwise. There, evidence abounds that humans are averse to estimation uncertainty – there called *ambiguity*. Ambiguity aversion often leads to fundamental inconsistencies in choices, as exemplified by the *Ellsberg Paradox* [14]. If anything, this suggests that estimation uncertainty enters valuation through a penalty.

We re-visited the choices generated by the restless six-arm bandit problem of [9] and investigated whether estimation uncertainty changed valuation positively (exploration bonus) or negatively (ambiguity penalty).

Finally, we studied to what extent the empirical support for Bayesian learning depended on the level of detail participants received regarding the outcome generating process. In [9], participants were fully informed about the structure of the bandit problem (risks could be different across bandits; probabilities jumped with differing frequency across bandits; and jumps occurred simultaneously for a number of bandits). They were ignorant only about the values of the parameters (outcome probabilities, jump frequencies, occurrence of jumps). As such, there was no “structural uncertainty” (or *Knightian* uncertainty as it is known in economics; [20]–[24]). In contrast, in [4], participants were naive about the task structure, so there was substantial structural uncertainty. There, participant choices reflected adjustment of learning rates to *average* unexpected uncertainty, suggesting that they had learned some aspects of the outcome generating process.

Here, we report new results that clarify to what extent *trial-by-trial* choices reflected Bayesian updating under structural uncertainty. We re-ran the six-arm restless bandit experiment, but we varied the amount of structural uncertainty. In one treatment, we told participants nothing about the outcome generating process. In another treatment, we informed the participants about everything except unexpected uncertainty. The third treatment was a replication of [9], to calibrate the findings.

## Results

### Formal Analysis of the Task

**Figure 1:**
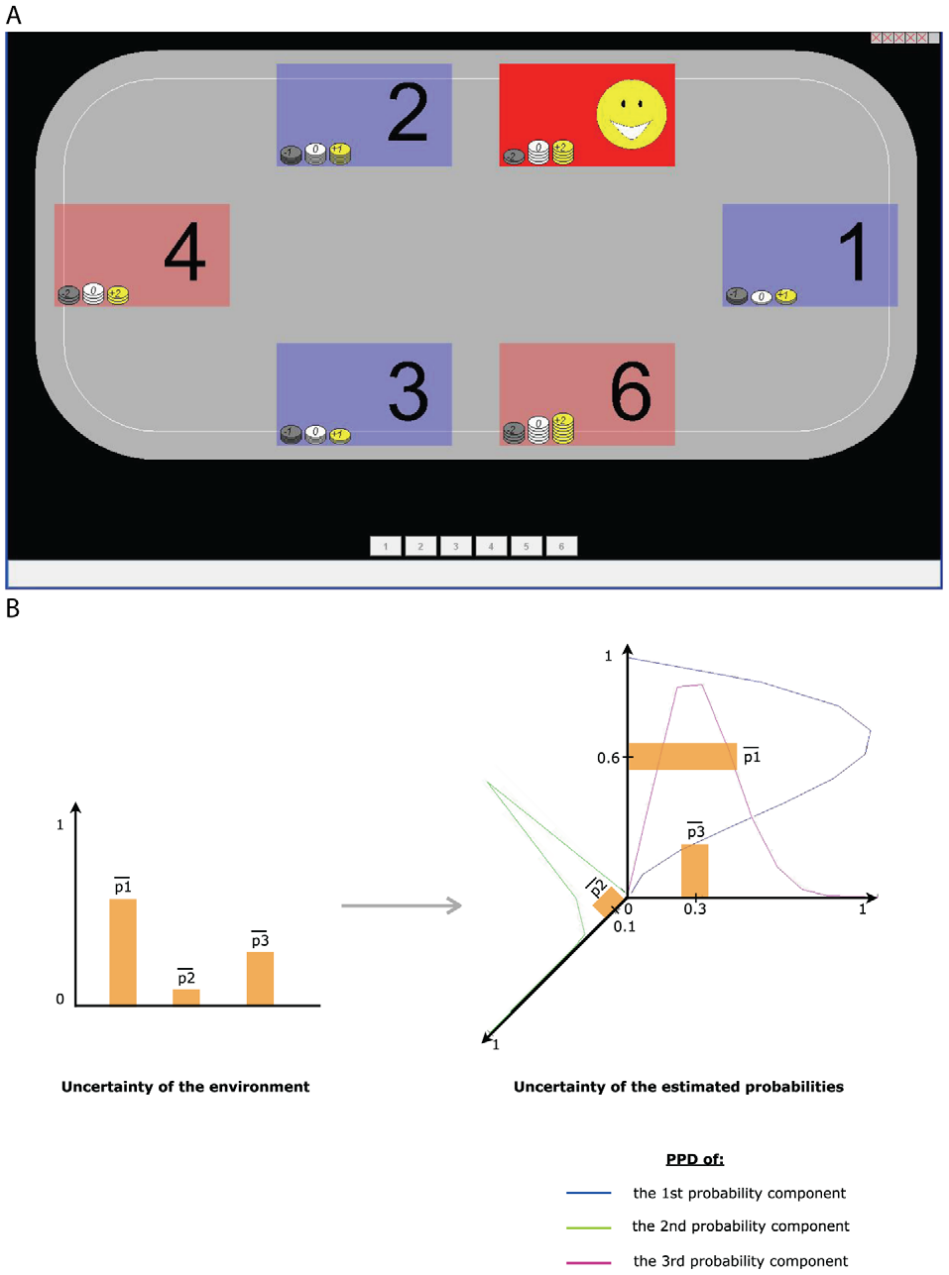
Six-arm restless bandit task. **A** The six-arm restless bandit is implemented graphically as a board game. Six locations correspond to the six arms. Locations are color-coded; blue locations have lower average unexpected uncertainty than red locations. Blue locations pay 1, 0 or −1 CHF (Swiss francs). Red locations pay 2, 0 or −2 CHF. Chosen option is highlighted (in this case, location 5). Participants can freely choose a location each trial. Histories of outcomes in locations chosen in the past are shown by means of coin piles. **B** Visual representation of risk and estimation uncertainty. Risk can be tracked using entropy, which depends on the relative magnitudes of the outcome probabilities, i.e., the relative heights of the bars in the left chart. The bars represent the three estimated outcome probabilities (mean of the posterior probability distribution or PPD). Entropy (risk) is maximal when the bars are all equal. Estimation uncertainty is represented by the widths of the posterior distributions of the outcome probabilities, depicted in the right chart.

Our task was a six-arm restless bandit problem, visually presented as a board game (see Fig. 1A). Arms were color-coded: the outcome probabilities for the red arms jumped more frequently. At each trial, arms paid one of three possible rewards: 1, 0 and −1 Swiss francs (CHF) for the blue arms, and 2, 0, −2 CHF for the red arms. Outcome probabilities were unknown.

Outcome probabilities within a color group jumped simultaneously. Participants did not know the jump frequencies. Nor did they know when jumps occurred. As such, there was unexpected uncertainty. After a jump, the outcome probabilities are given new, unknown values. Specifically, they did not revert to old values as in reversal learning tasks (e.g., [17]), and hence, there is estimation uncertainty throughout the duration of the task.

In the version of this task in [9], participants were fully informed about the structure of the outcome generating process. They merely had to learn (and, after each perceived jump, re-learn) the values of the outcome probabilities, as well as the probabilities of a jump (or the occurrence of a jump). We replicated this base version – to be referred to as Treatment 3 below. Additionally, we ran two variations of this board game, where we reduced the amount of structural information we gave the participants. We elaborate below. The three variations represent varying levels of model or structural uncertainty.

To analyze the results, we implemented a *forgetting Bayesian algorithm* [25] based on multinomial sampling under the Dirichlet prior with dynamic adjustment of the learning rate to evidence of the presence of jumps. In [9], a hierarchical Bayesian scheme was investigated as well. While qualitatively the same (and producing indistinguishable behavioral fits), the forgetting algorithm produces *explicit learning rates*, while in the hierarchical Bayesian approach, learning rates are only implicit. The availability of explicit formulae facilitated our analysis of the impact of the three levels of uncertainty on the learning speed.

In each trial 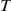, an option 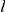 generated either the fixed loss outcome, denoted by 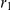, with probability 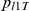, the null outcome (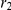), with probability 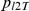, or the fixed reward outcome (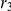), with probability 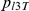. The triplet 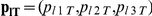 is in the three-dimensional simplex 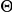; 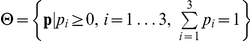.

We start from the same prior distribution of outcome probabilities for all options. It is denoted 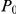. We take it to be an uninformative Dirichlet. At each trial 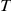 the Bayesian model updates the distribution of outcome probabilities based on a sufficient statistic that is constructed from the count vector 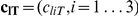, where 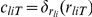. Here, 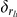 denotes point mass at 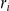. The forgetting algorithm takes the weighted geometric mean between the usual Bayesian update of the Dirichlet prior absent jumps and the original prior (for the case a jump occurred). Weighting is based on the subjective likelihood that no jump has occurred at trial 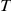, 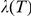 – more on the nature of 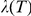 below. For large 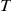, the resulting posterior is Dirichlet, like the prior. Specifically,
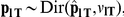


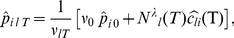
(1)

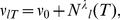
(2)where
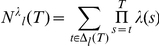
is the effective number of data used in the estimation of the outcome probabilities. Here, 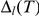 is the set of trials before (and including) trial 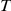 when option 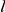 was chosen. The sufficient statistic 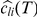 is defined as:
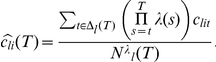
Significantly, this sufficient statistic can be obtained using simple recursive computations. Specifically, if option 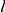 was chosen in trial 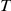,
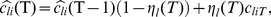
(3)where 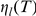, the learning rate, equals:
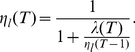
(4)The other case (when option 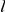 was not chosen in trial 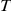) is discussed in the Methods.

In Eqn 3, 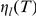 controls the relative weight of the new observation during learning. As such, it functions as the Bayesian learning rate. This is fortunate. Usually, the learning speed in Bayesian updating is only implicit; e.g., [4]. Because we have chosen to implement a forgetting algorithm, the speed of learning becomes explicit, in the form of a learning rate to be applied to the new observation.

The posterior mean outcome probabilities are computed as follows:
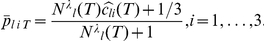
From these posterior means, the Bayesian decision maker computes the expected value (payoff) of option 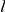, 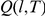.

To model adjudication between the six options, we opted for a softmax rule. Specifically, in trial 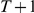, option 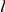 is chosen with probability
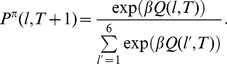
(5)Here, 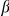 (also referred to as inverse temperature) measures the propensity to choose the option of currently greatest value rather than the others. As such, 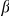 reflects the trade-off between the urge to exploit, i.e., to choose the best option, and the interest in exploring, i.e., to choose options currently deemed sub-optimal with the goal of learning more about their values [26], [27].

### Evolution of Uncertainty and Effect on the Learning Rate

[9] documents that in the current task, learning strategies behind human choices are better explained using Bayesian updating than (model-free) reinforcement learning, even if the learning rates in the latter are allowed to differ across choices with differing jump probability, or allowed to change over time as a function of the size of the reward prediction error. Crucial to correct setting of the Bayesian learning rate in our task is the ability to track three levels of uncertainty: risk, parameter estimation uncertainty, and unexpected uncertainty. The Bayesian model tracks these three levels independently, and they jointly affect the learning rate. We first illustrate their evolution, and then elaborate on how they modulate the learning rate.

Risk can be measured by the entropy of the outcome probabilities. Since outcome probabilities are unknown throughout our experiment, entropy needs to be estimated. We compute entropy based on the posterior mean of the outcome probabilities. See Fig. 1B for a graphical representation (left panel). Estimation uncertainty, on the other hand, reflects the spread of the posterior distribution of outcome probabilities. One could estimate it as the variance of the posterior distribution, or its entropy. See Methods for more information. Estimation uncertainty is depicted graphically in Fig. 1B (right panel). Unexpected uncertainty is the likelihood that outcome probabilities jump. Unexpected uncertainty changes over time, as evidence for jumps fluctuates. Average unexpected uncertainty differs also across options: blue locations on our board game have lower chance of jumping; red locations exhibit higher jump probabilities.

**Figure 2:**
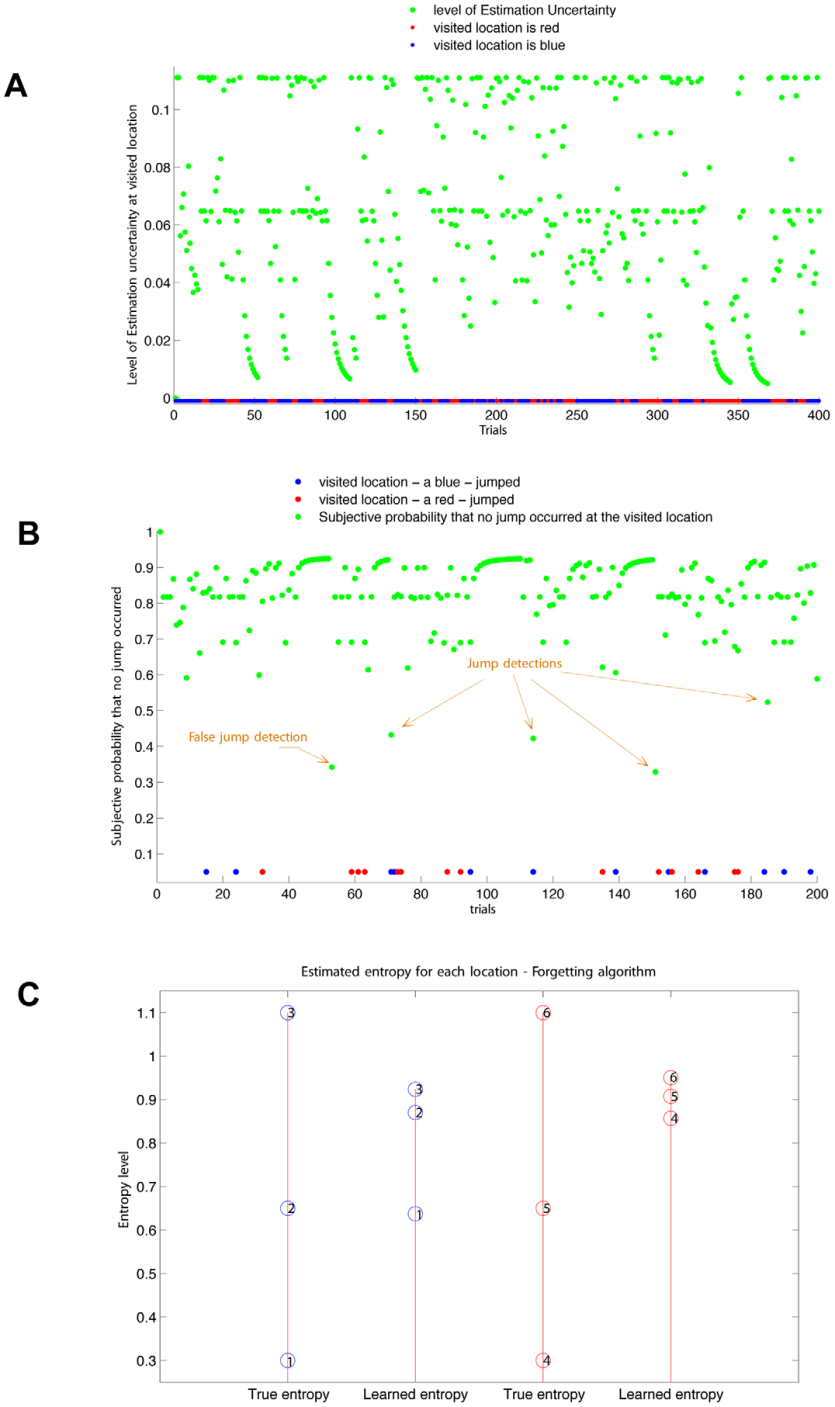
Three kinds of uncertainty in the task. **A** Evolution of the estimation uncertainty (entropy of mean posterior outcome probabilities) of chosen options in one instance of the board game. Learning is based on choices of one participant in our experiment. Blue dots on the horizontal axis indicate trials when a blue location was chosen; red dots indicate trials when a red location was visited. **B** Evolution of the unexpected uncertainty of chosen options in one instance of the board game, measured (inversely) as the probability that no jump has occurred. Learning is based on choices of one participant in our experiment. Blue dots on the horizontal axis indicate trials when outcome probabilities for the visited blue location jumped; red dots indicate trials when outcome probabilities for the visited red location jumped. **C** Average estimated risk (entropy of outcome probabilities) in one instance of the board game, by location (numbered 1 to 6). Learning is based on the choices of one participant in our experiment. Locations are arranged by level of unexpected uncertainty (blue: low; red: high). Average estimated risks are compared with true risks. The participant managed to distinguish risk differentials across blue locations, but not across red locations. Average estimated risks regress towards the grand mean because of estimation uncertainty after each jump in outcome probabilities.

Fig. 2A displays the evolution of estimation uncertainty in one instance of the task, based on choices of one participant. Estimation uncertainty is measured here at each trial as entropy of the posterior distribution of outcome probabilities. Estimation uncertainty is shown only for the chosen option. Blue dots indicate that an option was chosen with low average unexpected uncertainty (a blue location); red dots indicate choices of options with high average unexpected uncertainty (red locations). Estimation uncertainty increases each time the participant switches locations. The participant either switches to another location with the same color code (same average unexpected uncertainty) or to a location with a different color code.

Fig. 2B displays the evolution of the probability that no jump occurred in the first 200 trials of another instance. High levels indicate low levels of unexpected uncertainty. Low levels suggest detection of a jump, and hence, high unexpected uncertainty. Blue dots indicate trials when the chosen option was blue (low average unexpected uncertainty) and a jump in blue locations occurred simultaneously. Red dots indicate trials when the chosen option was red and a jump occurred (for the red options). At times, the participant seemed to have falsely detected a jump (e.g., in trial 54); but generally, the participant's belief that a jump has occurred correlates with actual occurrence of jumps.

The presence of unexpected uncertainty and the recurring parameter estimation uncertainty make it more difficult to correctly assess risk. Fig. 2C shows the mean level of risk assessed in one instance of the task. Bayesian updating is assumed. Shown are the average outcome entropies of each of the six options based on posterior mean probabilities. Options are numbered 1 through 6. For comparison, we also display the true outcome entropies. Results are stratified by level of average unexpected uncertainty: blue options had lower probability of jumps in outcome probabilities, while red options had high jump probabilities. The presence of high unexpected uncertainty affects learning of risk levels. On average, correct assignment of risk obtains for blue locations. But it is more difficult to correctly assess the risk of red locations.

The latter illustrates the *antagonistic relationship* [5] between the perceptions of unexpected uncertainty and risk. If the former is high, the latter is harder to estimate. A legitimate concern is, therefore, whether these two sources of uncertainty can be separately identified if participants are not told about their presence. We will elaborate below.

The different levels of uncertainty affect the learning rate in complex ways. Inspection of Eqn. 4 shows that the learning rate 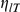 changes as a function of the ratio of the probability that no jump has occurred and the past learning rate:
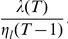
If the evidence for unexpected uncertainty is very low, i.e., if a jump is deemed unlikely, 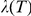 is close to 1, and hence, the learning rate decreases as in the absence of jumps, reflecting merely the decrease in estimation uncertainty. If, in contrast, the evidence for jumps is high, i.e., 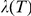 is close to zero, then the learning rate increases towards 1 irrespective of the past learning rate. This increase reflects the likely presence of a jump, and hence, the need to learn anew. That is, estimation uncertainty increases and so should the learning rate.

This shows how unexpected uncertainty affects estimation uncertainty, and hence, the learning rate. While not directly, estimation uncertainty itself does affect the learning rate, through its effect on unexpected uncertainty. This can be verified by inspecting the formula for the probability that no jump occurred in trial 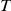:
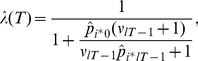
(6)where 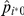 and 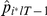 denote the estimated probability, initially and in trial 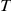 respectively, of observing outcome 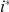 (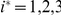, for loss, zero income, and gain, respectively) and where 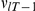 parameterizes the precision of the posterior distribution of outcome probabilities, which depends on the effective number of data used in estimating those outcome probabilities (see Eqn. 2). (See Methods for the derivation.) Estimation uncertainty, or its inverse, precision of the posterior distribution of outcome probabilities, therefore influences the estimate of the likelihood that no jump has occurred, and hence, unexpected uncertainty. In turn, unexpected uncertainty determines changes in the learning rate.

An analogous result obtains for risk – here defined as the entropy of the outcome probabilities. Intuitively, entropy is the variability in the probabilities across possible outcomes. If all outcome probabilities are the same, entropy is maximal. If one or more outcome probabilities are extreme (high or low), then entropy will be low. Eqn. 6 shows that unexpected uncertainty depends on outcome probabilities. The intuition is simple: if a particular outcome is estimated to occur with low probability, and that outcome does realize, the likelihood that it occurred because there was a jump is higher; conversely, if an outcome had high *a priori* probability, then its occurrence is unlikely to be attributed to unexpected uncertainty. Through its effect on unexpected uncertainty, estimated outcome probabilities have an effect on the learning rate.

Consequently, while the three levels of uncertainty separately influence the learning rate, unexpected uncertainty is pivotal. That is, estimation uncertainty and risk impact the learning rate through their effect on unexpected uncertainty. For instance, if the probability of an outcome is estimated with low precision (estimation uncertainty is high) or if it is estimated to be average (around 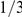), revealing high risk, then the realization of this outcome is unlikely to be attributed to a jump. The parameter 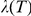 is therefore high, and the Bayesian learning rate 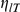 reduces as if one were in a jump-free world.

**Figure 3:**
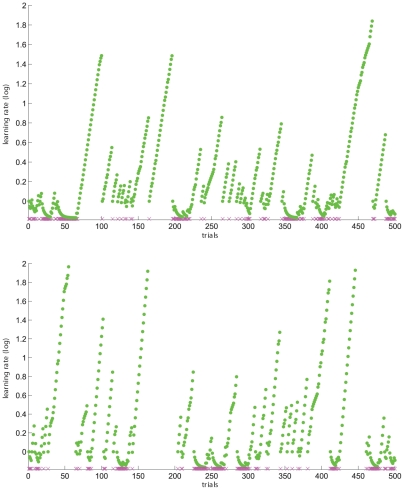
Evolution of the (logarithm of the) Bayesian learning rate for two options in one instance of the board game. Learning is based on the choices of one participant in our experiment. Top option has low average unexpected uncertainty (low chance of jumps) and low risk (one outcome probability was very high); bottom option has high average unexpected uncertainty and low risk. Crosses on the horizontal axis indicate trials when the option was chosen.

Fig. 3 displays the evolution of the learning rate for two options in one instance of the task. Shown are the (logarithm of) the learning rates of (i) an option with low risk and low average jump probability (low average unexpected uncertainty) [top], (ii) an option with low risk and high average jump probability [bottom]. The learning history is based on the actual choices of one of the participants in the experiment. Crosses on the horizontal axis indicate trials where the participant chose the option at hand.

One can easily discern the effect of a reduction in estimation uncertainty on the learning rate. During episodes when the participant chooses an option, she learns about the outcome probabilities, which reduces estimation uncertainty, and hence, the learning rate. This continues until she stops visiting the location at hand, and consequently, the – now imaginary – learning rate increases again. (We call the learning rate “imaginary” because there are no outcomes to be used to update beliefs; belief updating for the unchosen options evolve only because of what one learns about the chosen options.)

### Exploration Bonuses and Ambiguity Penalties

To implement Bayesian learning, the decision maker has, at a minimum, to track estimation uncertainty. As such, the decision maker senses that she does not know the parameters, and hence, she is ambiguity sensitive.

In multi-armed bandit settings, exploration is valuable. Only by trying out options will one be able to learn, thus reducing estimation uncertainty. As such, there should be a bonus to exploration of options with high ambiguity. This was recently proposed [18], [28]. Decision makers should therefore be ambiguity seeking, which conflicts with claims that humans generally exhibit ambiguity aversion [14], [19].

Here, we re-visit behavior in our six-arm restless bandit task to determine to what extent choices reflect the presence of an exploration bonus or an ambiguity penalty, both equal to the level of estimation uncertainty. To this end, we added to the expected value of each option an exploration bonus, or alternatively, we subtracted an ambiguity penalty – computational details are provided in Methods. For each participant, we compared the maximum log-likelihood of the model with exploration bonus to that of the base version of the Bayesian model; likewise, we compared the log-likelihood of the model with ambiguity penalty to that of the base model. The log-likelihood of a model is defined by Eqn. 8 in Methods.

**Figure 4:**
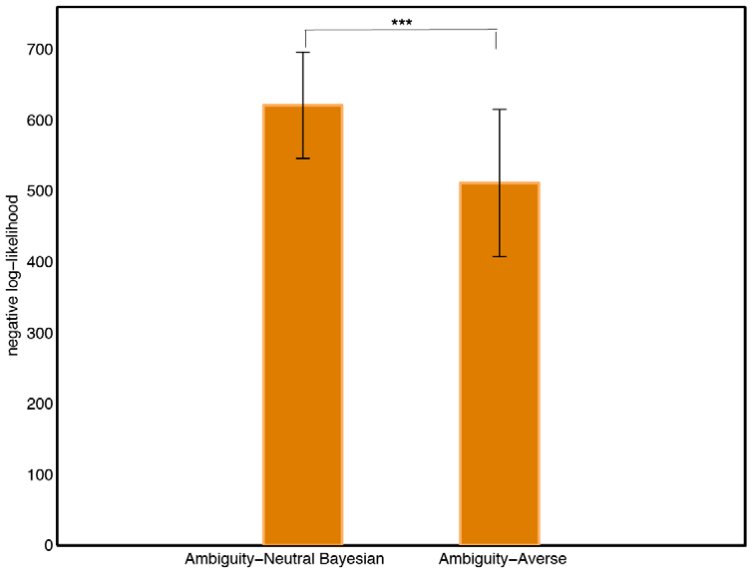
Goodness-of-fits of the Bayesian models, with (right) and without (left) penalty for ambiguity. Based on approximately 500 choices of 62 participants. Data are from [9]. Heights of bars indicate mean of the individual negative log-likelihood; line segments indicate standard deviations. 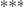: 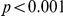; 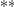: 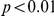; 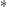: 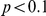.

The model with exploration bonus fitted worse than the one without any correction of valuations for estimation uncertainty. In contrast, the model with ambiguity penalty generated a better likelihood than did the base version of the Bayesian model for 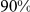 of the participants. The individual log-likelihoods are reported graphically in Fig.S1 of the Supporting Information. Fig. 4 displays the mean negative log-likelihoods and the corresponding sample standard deviations across the 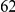 subjects. A *paired t-test* based on the difference between the log-likelihoods of the two models (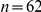) leads to reject the hypothesis that this difference is null with a p-value equal to 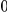.

### Structural Uncertainty

To investigate to what extent the evidence in favor of Bayesian updating is related to our providing subjects with ample structural knowledge of the outcome generating process, we ran a new experiment. We considered three treatments. In the first treatment, we provided subjects only with the rules of the game, and no structural information. In the second treatment, subjects were given some structural information (e.g., within a color group, one option was “biased” in the sense that its entropy was lower, while another option was close to random), but were left ignorant about the presence of jumps in the outcome probabilities; which means they were not informed about the potential of unexpected uncertainty. The third treatment was a replication of the original setting in [9].

43 undergraduates from the same institution (Ecole Polytechnique Fédérale Lausanne) participated in the first treatment; 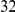 (30) of them participated in the second (third) treatment. (We presented the three treatments as three separate experiments, whereby participants in the first treatment were invited but not forced to participate in the two others.)

**Figure 5:**
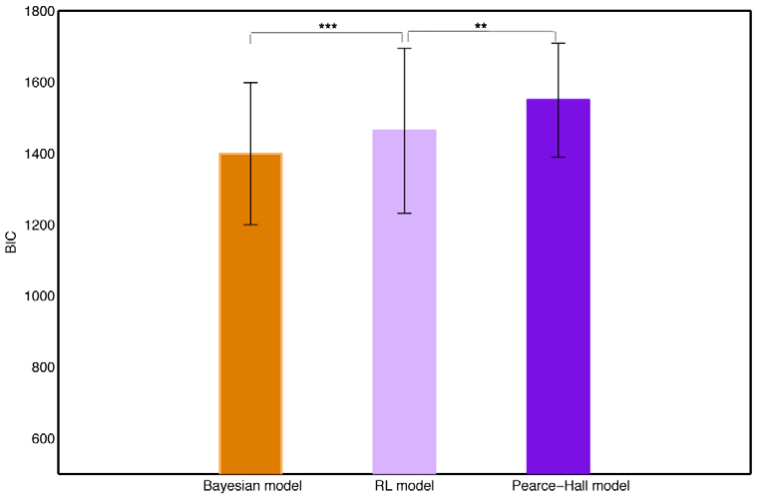
Replication of the experiment in [9]. Mean BICs and standard deviations of the Bayesian, reinforcement and Pearce-Hall learning models without structural uncertainty (Treatment 3). Based on the choices of 30 participants in approximately 500 trials of our board game. The Bayesian model is the base version (unadjusted for ambiguity aversion). 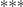: 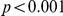; 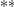: 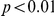; 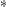: 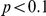.

To calibrate the results, we first compare the fits of the third treatment to those of [9]. Like in [9], we compare the log-likelihood of the base version of the Bayesian model to the one of a Rescorla-Wagner rule in which the learning rates are allowed to differ across choices with differing jump probability (henceforth, the “reinforcement learning model”), and also to the one of the Pearce-Hall extension of reinforcement learning. Fig. 5 displays the mean BIC across the 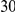 participants for each of the three models – the BIC or *Schwarz Criterion* [29] of a model is the negative log-likelihood corrected for differences in number of parameters to be estimated. Corresponding sample standard deviations are also reported. A paired t-test based on the difference between the BICs of the Bayesian and reinforcement learning models (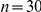) leads to the conclusion that the Bayesian model fitted better than the reinforcement learning model with a p-value smaller than 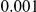. Like in [9], the Pearce-Hall model fitted the data worst. The finding that the model with ambiguity penalty provided the best fit is also replicated. The distributions of the individual log-likelihoods for all four models (the base version of the Bayesian model, the version with ambiguity penalty, the reinforcement learning model and the Pearce-Hall extension) are available in the Supporting Information (see Fig.S2).

**Figure 6:**
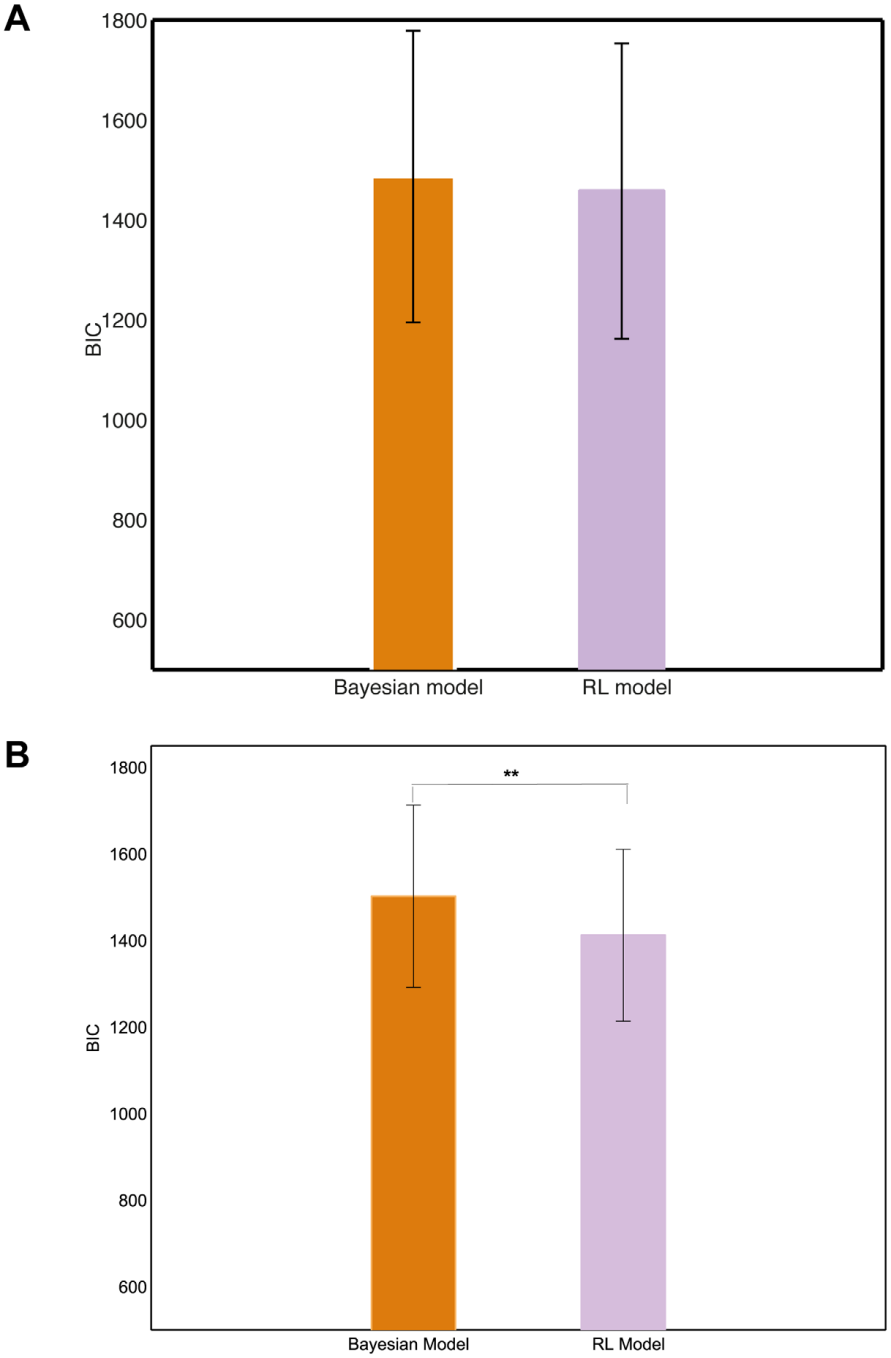
Goodness-of-fits of the Bayesian and reinforcement learning models under varying levels of structural uncertainty. **A** Goodness-of-fits of the Bayesian and reinforcement learning models under full structural uncertainty (Treatment 1). Based on the choices of 43 participants in approximately 500 trials of our board game. The Bayesian model includes a penalty for estimation uncertainty – like in the data from [9], this model turned out to fit the data better than the base version of the Bayesian model. Heights of bars indicate mean of the individual Bayesian Information Criterion (BIC); line segments indicate standard deviations. The difference in the mean BIC is not significant (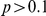). **B** Goodness-of-fits of the Bayesian and reinforcement learning models under partial structural uncertainty (Treatment 2). Mean BICs and standard deviations of the Bayesian and reinforcement learning models in Treatment 2. Based on the choices of 32 participants in approximately 500 trials of our board game. The Bayesian model includes a penalty for estimation uncertainty. Participants knew the structure of the game except for the jumps in outcome probabilities. They were told that the description of the structure was incomplete. 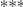: 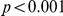; 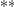: 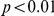; 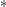: 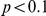.

Having replicated the results with full disclosure of the structure of the outcome generating process, we turn to the first treatment, where subjects were not given any structural information. Fig. 6A compares the mean BIC of the Bayesian model with ambiguity penalty – which appeared to outperform the base Bayesian model in all treatments – to the one of the reinforcement learning model. Corresponding sample standard deviations are displayed as well. The fit of the ambiguity averse Bayesian model now does not improve any more upon simple reinforcement learning, according to a paired 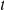-test based on the difference between the BICs of the two models (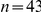, 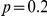). In the second treatment, the reinforcement learning model marginally outperformed the ambiguity averse Bayesian model: a paired 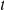 test (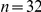) leads to the conclusion that the reinforcement learning model fitted better with a p-value equal to 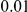. See Fig. 6B. In both treatments, the fit of the Pearce-Hall model was worst for the large majority of the subjects, and we do not report it on Fig. 6A or Fig. 6B. The distributions of the individual log-likelihoods of all models are reported in the Supporting Information (see Fig.S3 and Fig.S4).

Common to both Treatments 1 and 2 is the absence of information on the presence of unexpected uncertainty. The findings suggest that participants were not able to recognize that outcome probabilities jumped. To verify this conjecture, we examined the answers to the debriefing questionnaire after the experiment – participant answers are available upon request. Pooling the first two treatments (with a total of 75 cases), only 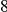 participants detected the presence of instability (they realized that for certain of the six arms, “dark periods” alternated with good ones during the task). When asked whether it would be “equally difficult to learn on the red locations and the blue ones,” many subjects answered affirmatively, despite the fact that the probability of a jump (in outcome probabilities) on the red locations was four times higher. A typical case was that of a participant in the second treatment who reported: “At some point I got several bad outcomes but I tried to be rational and stay since it was the good one.” The participant mis-attributed a sequence of bad outcomes to risk, rather than interpreting it as evidence for a regime shift.

These findings are significant. In no way did the instructions attempt to mislead the participants. On the contrary, we stated explicitly that subjects had to watch out for features of the outcome generating process other than those spelled out in the instructions. In contrast, in the third treatment (as well as in the original experiment of [9]), responses on the debriefing questionnaire indicated that participants managed to detect changes during the task, and could often estimate quite accurately the relative jump probabilities across location colors.

## Discussion

### Neural Evidence for Separate Encoding of Uncertainty Levels

On occasion, humans have been shown to choose like Bayesian decision makers. In a context where outcome contingencies change constantly, this implies that humans should be able to distinguish various types of uncertainty, from unexpected uncertainty, over (parameter) estimation uncertainty, to risk. We will argue here that there exists emerging neurobiological evidence for separate encoding of these categories of uncertainty. As such, key components for neural implementation of Bayesian learning have become identified in the human brain.

Numerous studies have localized neural signals correlating with risk. Some sub-cortical regions are also involved in tracking expected reward (striatal regions; [30]) and the relatively crude fMRI evidence is supported by single-unit recordings in the monkey brain [31]; the evidence for neural signals of risk independent of expected reward has been identified mostly in cortical structures (anterior insula, anterior cingulate cortex, inferior frontal gyrus, and interparietal sulcus) [11], [12], [30], [32]–[35].

Estimation uncertainty, or ambiguity as it is referred to in economics, has also recently been investigated in imaging studies. Early evidence pointed to involvement of the amygdala and lateral orbitofrontal cortex [15]; subsequent parametric work has corroborated [12] and extended with activation of the frontopolar cortex [3]. Experimental paradigms where estimation uncertainty is manipulated as in the six-arm restless bandit problem have yet to be organized.

Involvement of locus coeruleus and the neurotransmitter norepinephrine in tracking unexpected uncertainty has been conjectured a number of times and the evidence in its favor is suggestive [5], [28,28], but further proof is needed. Unexpected uncertainty will have to be manipulated parametrically, as norepinephrine is known to be generally involved in attention modulation as well as general exploratory behavior [36]. Without parametric manipulation, activations can as well be interpreted as reflecting attention or exploration.

Activation of the amygdala-hippocampus complex to novel images in a learning context may be conjectured to reflect unexpected uncertainty [37], [38]. Neural correlates with the Bayesian learning rate have been identified in the precuneus and anterior cingulate cortex [4], [39]. Because of the close relationship between the Bayesian learning rate and unexpected uncertainty (effects of risk and estimation uncertainty on the learning rate operate through unexpected uncertainty, as explained before), these neural signals could as well reflect unexpected uncertainty (changes in the likelihood that outcome probabilities have jumped).

### Bayesian Exploration

Evidence has thus emerged that the distinction of the three forms of uncertainty exists at the neuronal level. The well-documented sensitivity of humans to ambiguity (estimation uncertainty) further proves that the distinction can readily be observed in behavior. Confirming humans' sensitivity to estimation uncertainty, we presented evidence here that participants' tendency to explore in a six-arm restless bandit task decreased with estimation uncertainty. This finding falsifies the hypothesis that estimation uncertainty ought to induce exploration. It is, however, consistent with evidence of ambiguity aversion in the experimental economics literature, starting with [14], [19]. We are the first to show the parametric relationship between estimation uncertainty and exploration: the relationship is negative.

The reader may wonder why we have not augmented the reinforcement learning model with an ambiguity penalty, and examined the behavioral fit of this version of model-free reinforcement learning. The point is that non-Bayesians do not sense ambiguity. Indeed, the concept of a posterior belief is foreign to non-Bayesian updating, and hence, the variance or entropy of the posterior distribution of outcome probabilities, our two measures of estimation uncertainty, are quintessentially Bayesian. Since the representation of ambiguity is absent in the context of model-free reinforcement learning, a fortiori ambiguity cannot weigh in the exploration strategy. In light of this, one should not combine model-free reinforcement learning with an ambiguity penalty/bonus.

### Ambiguity vs. Structural Uncertainty

A third major finding was that full Bayesian updating is reflected in human learning only if enough structural information of the outcome generating process is provided. Specifically, the ability to track unexpected uncertainty, and hence, to detect jumps in the outcome probabilities, appeared to rely on instructions that such jumps would occur. When participants were not informed about the presence of unexpected uncertainty, their choices could equally well be explained in terms of simple reinforcement learning. This evidence emerged despite suggestions to watch for features of the outcome generating process that were not made explicit in the instructions.

Situations where decision makers are ignorant of the specifics of the outcome generating process entail model or structural uncertainty. Our study is the first to discover that humans cannot necessarily resolve model uncertainty. In our experiment, many participants failed to recognize the presence of unexpected uncertainty. Consequently, in the exit questionnaires they often took the arms to be “random” [in our language, risky] which illustrates the antagonistic relationship between risk and unexpected uncertainty – jumps were confounded with realization of risk.

Our participants' failure to detect jumps may suggest that their “mental models” excluded nonstationarity *a priori*. Mental models are expectancies or predispositions which serve to select and organize the information coming from the environment [40], [41]. *Nudging* [42] may be needed, whereby the instructions bring the likely presence of jumps to the attention of the participants.

Structural uncertainty was originally suggested in the economics literature, where it is referred to as *Knightian* or *Keynesian* uncertainty [20], [21]. Nevertheless, even in economics, structural uncertainty is often treated interchangeably with estimation uncertainty or ambiguity; e.g., [43]. In principle, structural uncertainty can be dealt with by introducing extra parameters that identify the possible models of the outcome generating process. Structural uncertainty thereby collapses to simple (parameter) estimation uncertainty.

Nevertheless, we think it is important to refrain from reducing structural uncertainty to mere parameter estimation uncertainty, because the number of possible models of the outcome generating process in any given situation is large, and hence, the number of parameters to be added to capture structural uncertainty can be prohibitively high [24]. It is well known that Bayesian updating will fail dramatically when the parameter space is high-dimensional [44]; in such situations, model-free reinforcement learning produces, in a simple and consistent way, the right statistics to guide choice.

The latter may explain our finding that human choice in our six-arm restless bandit task reveals less evidence of Bayesian updating when we introduce structural uncertainty. Since reinforcement learning provides ready guidance in situations where Bayesian updating may fail, our participants understandably switched learning strategies. Because they became (model-free) reinforcement learners, they no longer detected unexpected uncertainty. Indeed, uncertainty is monolithic in the absence of a model of the outcome generating process; there is no distinction between risk, estimation uncertainty, unexpected uncertainty, or even model uncertainty.

To conclude, our results suggest that learning-wise, structural uncertainty should not be thought of as an extension of ambiguity. We thus advocate a separation of situations entailing structural uncertainty and situations entailing ambiguity in future studies of decision making under uncertainty. We would also advocate a clear separation of situations where the outcome probabilities change suddenly and the related but mathematically distinct situations, where outcome probabilities change continuously. The former entail unexpected uncertainty. The latter are analogous to the contexts where Kalman filtering provides optimal forecasts, but where risk is stochastic. In financial economics, one therefore uses the term *stochastic volatility* [45]. Recently, computational neuroscientists have underscored the need to distinguish between unexpected uncertainty and stochastic volatility [46].

In our six-arm restless bandit, the three levels of uncertainty change in equally salient ways. Future imaging studies could therefore rely on our task to better localize the encoding of uncertainty and its three components. In addition, our task could allow one to investigate engagement of brain structures in the determination of the learning rate.

## Methods

### Ethics Statement

All the experiments reported on here had the approval from the ethics commission of the Ecole Polytechnique Fédérale Lausanne.

### The Task

We implemented a six-arm restless bandit task with a board game. See Fig. 1A. Participants played approximately 500 trials of this game. We investigated learning behind participants' choices from two experiments. The data from the first experiment were originally presented in [9]. In this experiment, participants were given precise instructions about the structure of the outcome generating process. That is, there was no structural uncertainty. In the second experiment, we invited new participants to play our board game, under one of three treatments. In Treatment 1, participants were not told anything about the structure of the outcome generating process. That is, there was full structural uncertainty. In Treatment 2, participants were told everything about the outcome generating process except the presence of jumps. Participants were warned that the structural description was not complete, and were invited to pay attention to possible structure beyond that revealed in the instructions. Treatment 3 was a replication of the experiment in [9] – as such, there was no structural uncertainty.

### Bayesian Learning in the Task

In our Bayesian learning model, the distribution of outcome probabilities is updated using Bayes' law and a *stabilized forgetting* [25] operator. At trial 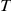, Bayes' law transforms the given prior to the posterior using the likelihood of the observed outcome and the prior. The transformation depends on a sufficient statistic which is constructed from the count vector 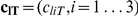, where 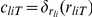. Here, 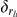 denotes the point mass at 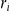 (i.e., 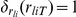 if the outcome at location 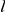 in trial 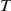 equals 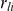, and 0 otherwise).

Since our task involves multinomial outcomes, we chose a Dirichlet prior to initiate learning. Without jumps, posterior distributions will be Dirichlet as well. As initial (first-trial) prior, we take the uninformative Dirichlet with center 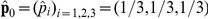 and precision 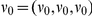 where 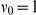. Formally, the Dirichlet prior equals:
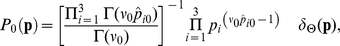
where 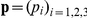, 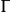 is the Gamma function (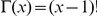) and 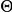 denotes the three-dimensional simplex, i.e., 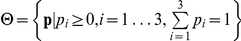.

Let 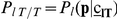 denote the posterior distribution absent jumps. It is obtained from the prior in the usual way, by combining the prior with the (multinomial) likelihood of the count vector 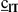. The posterior is Dirichlet as well, like the prior.

In a stationary world, this would provide the optimal inference. Because jumps may occur (outcome probabilities may change), we augment the standard Bayesian updating using a forgetting operator, which we denote 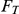.


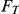
 combines two distributions to generate a new posterior, 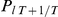. These two distributions are the following.

- After a jump in trial 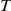, the posterior should no longer be 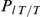, but another reference probability distribution. Here, we use 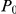, the initial prior.
- In the absence of a jump, the decision maker should use the standard Bayesian posterior, here denoted 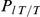.

Therefore, in principle, the new posterior should either be 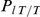, when there is no jump, or 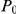, when there is one. But the decision maker does not observe jumps directly, and hence, has to weight the two cases based on the evidence for a jump. Our forgetting operator thus mixes the two possibilities:
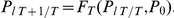



From minimization of a Bayes risk criterion, 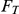 has to be taken to be a *weighted geometric mean* (see [9]). That is, 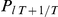 is the (weighted) geometric mean of 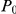 and 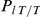. The weight depends on the estimate of the likelihood that a jump has not occurred, 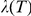. (Note that 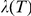 depends on the color of the location only, as all options within a same color category jump simultaneously.) The complement of 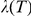, 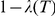, is a measure of jump likelihood, and hence, unexpected uncertainty.

Consequently, the forgetting operator equals:
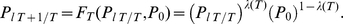



The geometric mean is a tractable way to introduce information on unexpected uncertainty in the updating because, for large 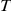, the posterior probability distribution is well approximated by a Dirichlet distribution, so that updates remain in the same family of distributions as the priors, namely, the family of Dirichlet priors. The proof is available upon request.

Another advantage of the forgetting operator, important for our purposes, is that updating can be expressed directly in terms of a learning rate. Usually, with Bayesian updating, learning rates are only implicit (because the Bayes transformation is generally non-linear). We shall use the symbol 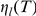 for the learning rate for option 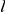 in trial 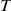.

Specifically, with the forgetting algorithm, the posterior mean probability vector is computed as follows:
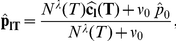
where 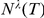, the effective number of observations used to update beliefs for location 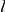, equals
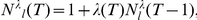
if location 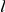 was chosen in trial 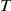, and otherwise:
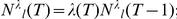
and where 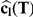 is a sufficient statistic based on past observed outcomes for location 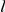, and updated as follows:
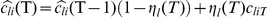
if option 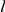 was chosen in trial 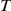, and
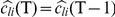
if not.

The learning rate 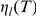 determines the weight on the most recent observation in the updating equation for the sufficient statistic 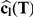. It is defined, recursively, as follows: if location 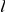 is chosen in trial 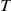, then
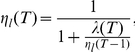
otherwise
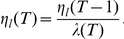
One can express the learning rate non-recursively:
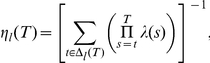
where the set 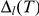 contains the trials up to 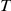 when location 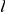 was visited.

### Model-Free Reinforcement Learning

For model-free reinforcement learning, we applied a simple Rescorla-Wagner rule. Let 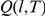 denote the value of option 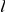 after the outcome in trial 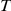.

- If 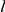 is sampled at trial T,
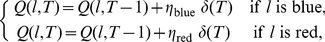
(7)where 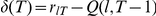 is the prediction error (outcome 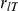 minus prediction).
- If 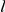 is not sampled at trial 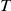, then 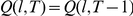.

Here, the learning rate is fixed but color-specific. As such, the reinforcement learning model allows for adjustment of the learning rate to the average level of unexpected uncertainty (red options jump more often than blue ones), in line with evidence that the learning rate increases with average unexpected uncertainty [4]. We also tested model-free reinforcement learning with a single learning rate across choices. The fit was worse, even after penalizing the model with dual learning rates for the extra degree of freedom.

We also fit a modified reinforcement learning model, where the learning rate adjusts to the size of the prediction error in the last trial. This is the Pearce-Hall model [8].

### Computation of Unexpected Uncertainty in the Bayesian Model

The computations, which are provided in [9], and available in Text S1. We repeat the key arguments here, for ease of reference. At each trial, the Bayesian decision maker needs to infer whether a jump has occurred. Since jumps are color-dependent only, the Bayesian model extrapolates such inference to all options with the same color as the chosen one. As before, 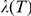 denotes the probability that no jump has occurred. 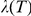 is color-specific and we shall write 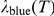 for the blue options and 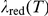 for the red ones. Without loss of generality, take 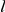, the visited location at trial 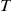, to be red. (In the main text, and earlier in the Methods Section, we dropped the color reference, to avoid unnecessary notational burden.) Formally,
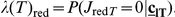
The computation of this subjective probability leads to
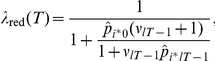
where 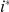 refers to the realized component of the count vector at time 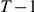. (For example, suppose that location 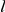 delivered the loss outcome at trial 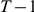; then 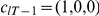, and 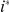 is equal to 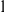.) Thus, 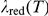 depends on 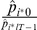, the *strength of evidence* for the hypothesis that a jump has occurred at time 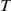.

Unexpected uncertainty, the chance that a jump has occurred, is complementary to the chance that no jump has occurred. At the red location, it equals 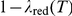. Therefore, 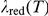 tracks unexpected uncertainty at the red location.

### Computation of Estimation Uncertainty in the Bayesian Model

Estimation uncertainty is the dispersion of the posterior distribution of outcome probabilities. It can be measured either by the variance or the entropy.

The *variance* metric for option 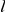 at trial 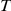 is computed as follows:
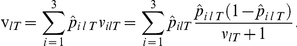
From [47], we define the *entropy* of the posterior probability distribution for option 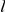 at 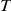 as follows:
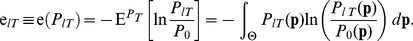
The entropy metric is thus
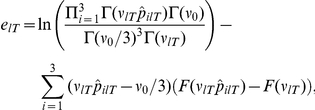
where 
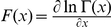
 is the Digamma function.

### Choice Model

We used the softmax function to transform valuations for the options into choice probabilities. It generated a probability distribution 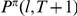 that location 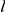 would be visited in the subsequent trial 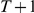. In the base version, valuations remained unadjusted, namely, the expected payoff in the next trial 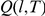. The softmax function depended on one parameter, namely, the inverse temperature 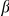. See Eqn. 5.

A couple of alternative versions were considered, by taking the average of the expected payoff and a bonus or (if negative) a penalty. The bonus/penalty was equal to the level of parameter estimation uncertainty (variance or entropy of the posterior distribution as defined above). In the model with bonus, 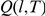 in Eqn. 5 was replaced with either 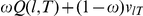 (when measuring estimation uncertainty with the variance metric) or 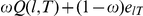 (when using the entropy metric). In the model with penalty, it was replaced with 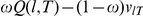 or 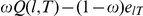. Without loss the parameter 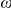 can be set equal to 1/2. This particular value is not pivotal in the sense that replacing it with 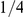 or 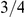 does not change the main results qualitatively (i.e., whatever the value of the parameter, the version with penalty significantly improved the fit of the base model, and the version with bonus did not).

### Model Fitting

Using participant choices, we fitted the free parameters of each model: 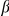 for the Bayesian and Pearce-Hall learning models; 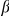, 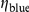 and 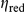 for the reinforcement learning model. For each participant, best fit was obtained by maximizing the log-likelihood 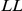 compounded over trials:
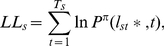
(8)where 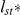 is the option chosen by subject 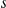 in trial 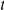, and 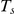 is the number of trials participant 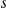 played.

## Supporting Information

Figure S1 Graphical display of the individual (negative) log-likelihoods of the Bayesian models, with penalty for ambiguity (Y-axis) and without (X-axis).
(0.01 MB PDF)

Figure S2 Graphical display of the individual (negative) log-likelihoods of the models in Treatment 3.
(0.02 MB PDF)

Figure S3 Graphical display of the individual (negative) log-likelihoods of the models in Treatment 1.
(0.02 MB PDF)

Figure S4 Graphical display of the individual (negative) log-likelihoods of the models in Treatment 2.
(0.02 MB PDF)

Text S1 Supplemental material.
(0.20 MB PDF)
