# Supplementary figures and images for: Risk, Unexpected Uncertainty, and Estimation Uncertainty: Bayesian Learning in Unstable Settings
Source: PLoS Comput Biol. 2011 Jan 20;7(1):e1001048. doi: 10.1371/journal.pcbi.1001048 (PMC3024253; doi:10.1371/journal.pcbi.1001048)

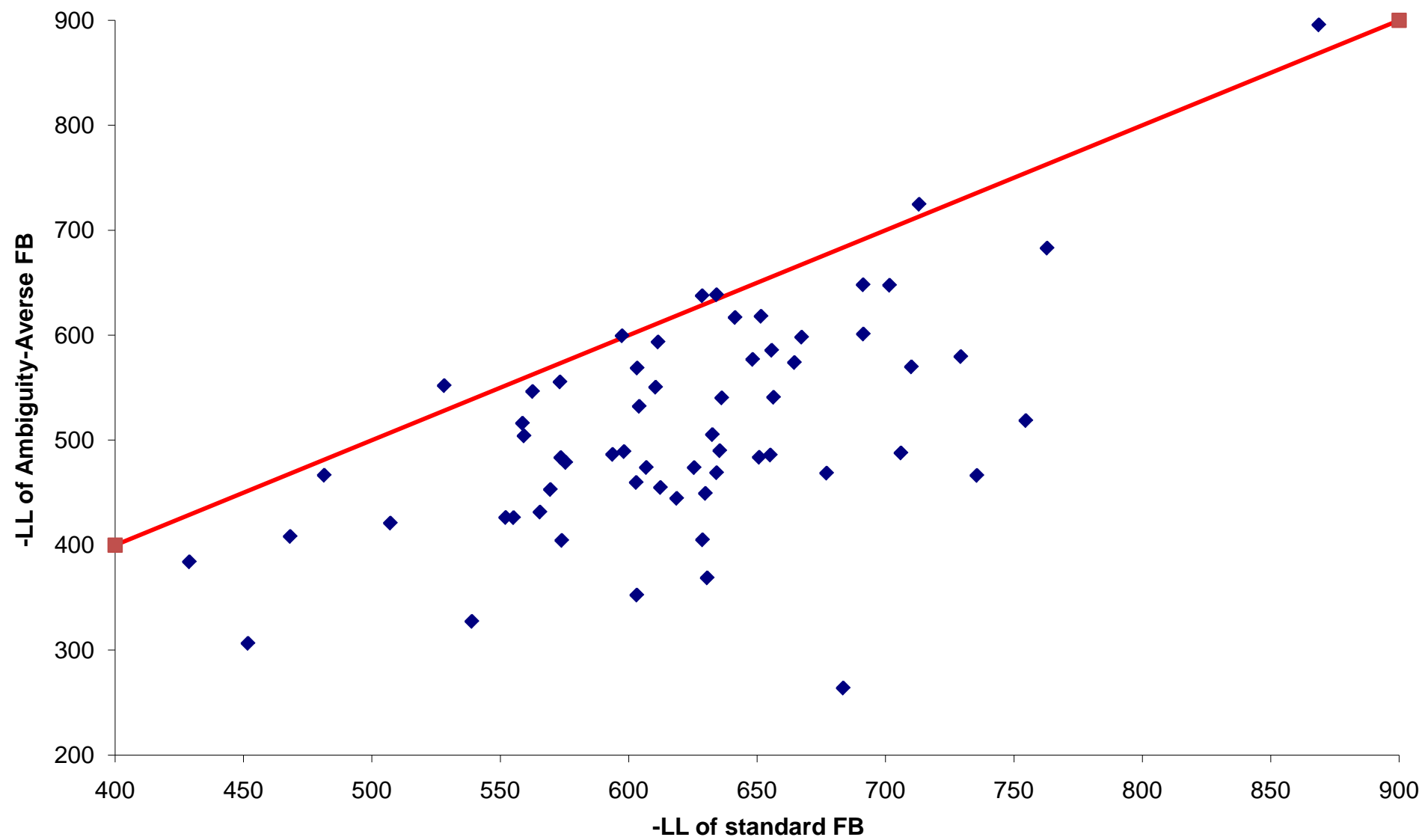

Supplement: Figure S1 — Graphical display of the individual (negative) log-likelihoods of the Bayesian models, with penalty for ambiguity (Y-axis) and without (X-axis). (0.01 MB PDF) [file pcbi.1001048.s001.pdf]

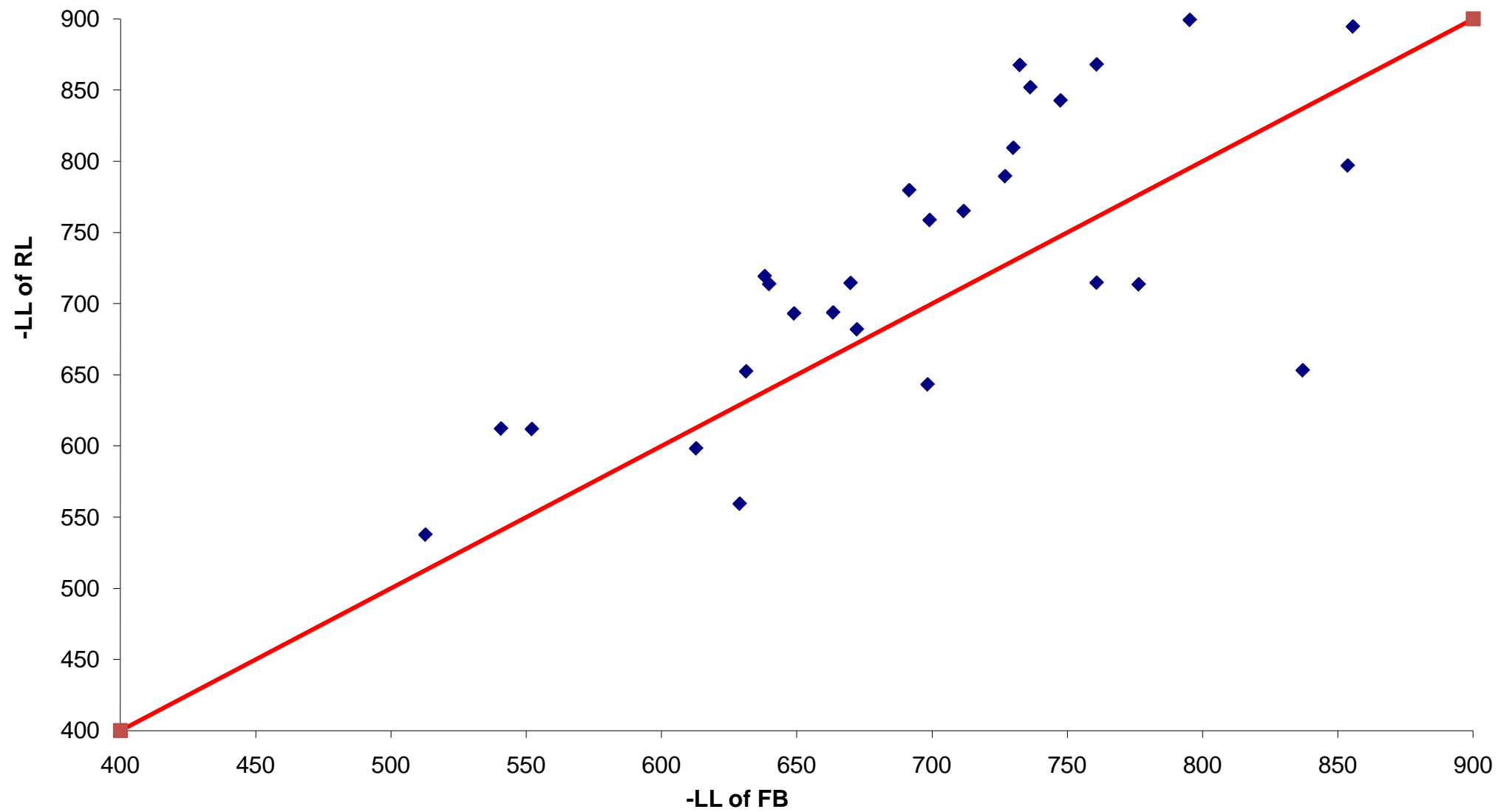

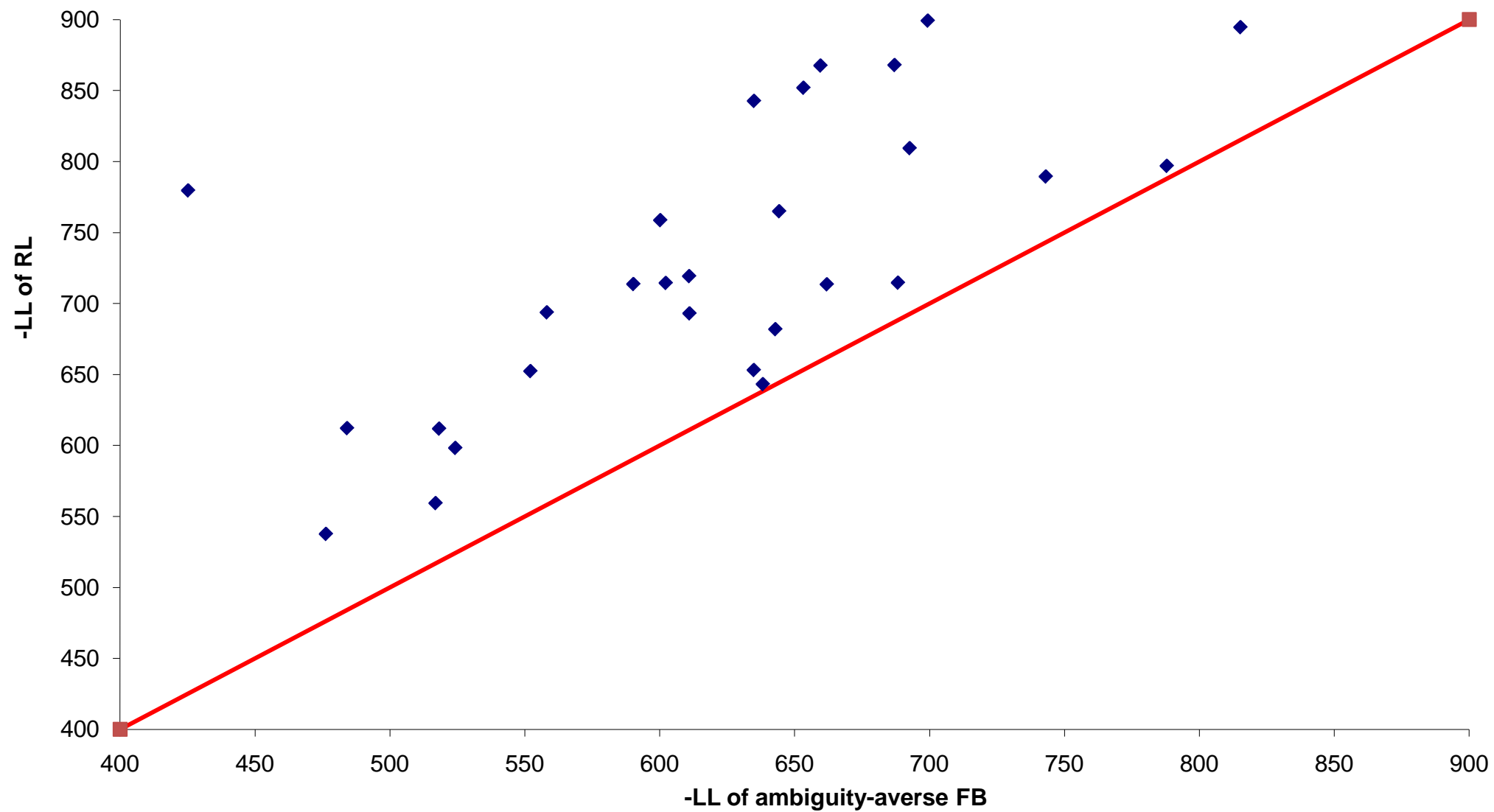

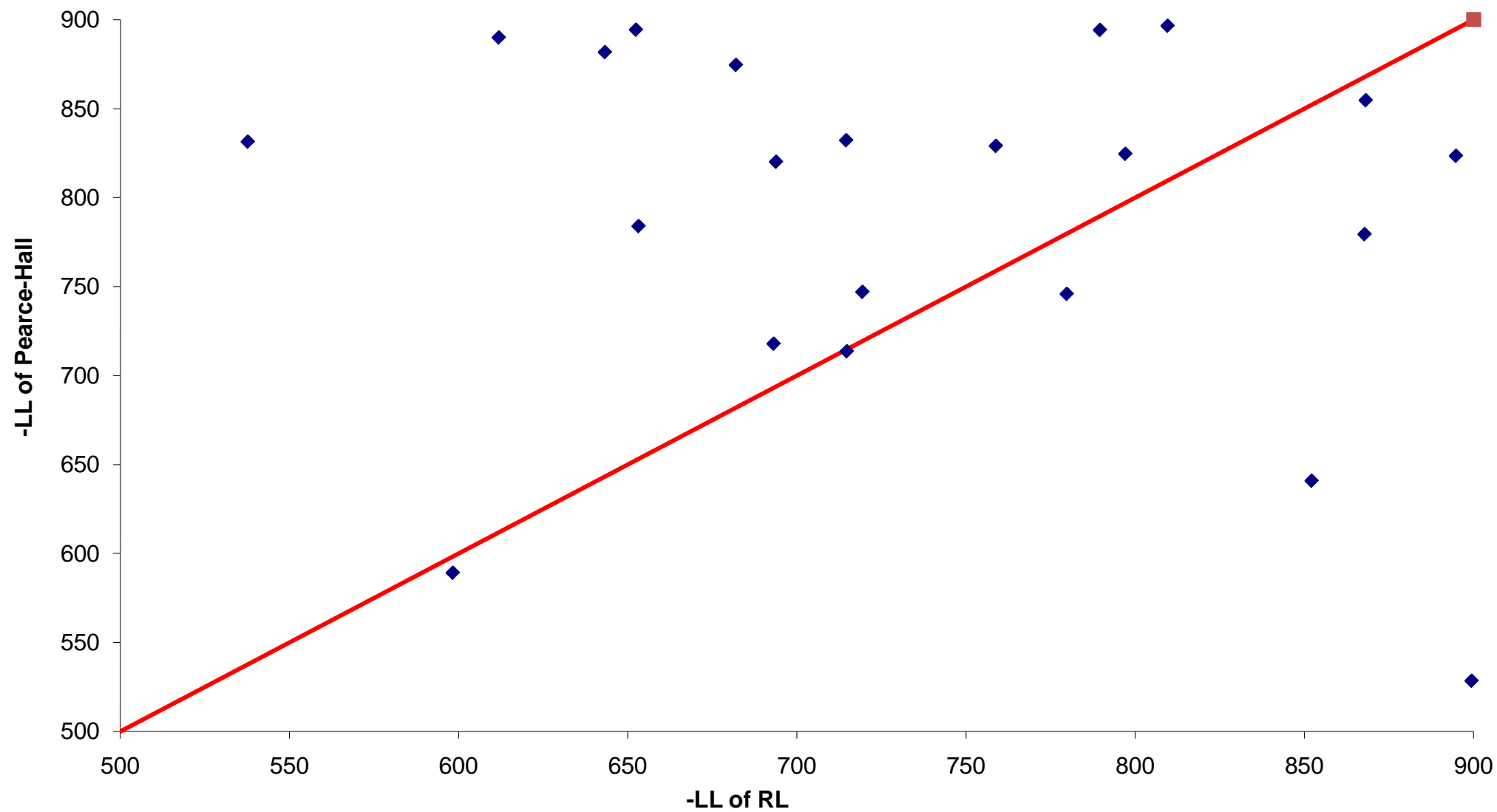

Supplement: Figure S2 — Graphical display of the individual (negative) log-likelihoods of the models in Treatment 3. (0.02 MB PDF) [file pcbi.1001048.s002.pdf]

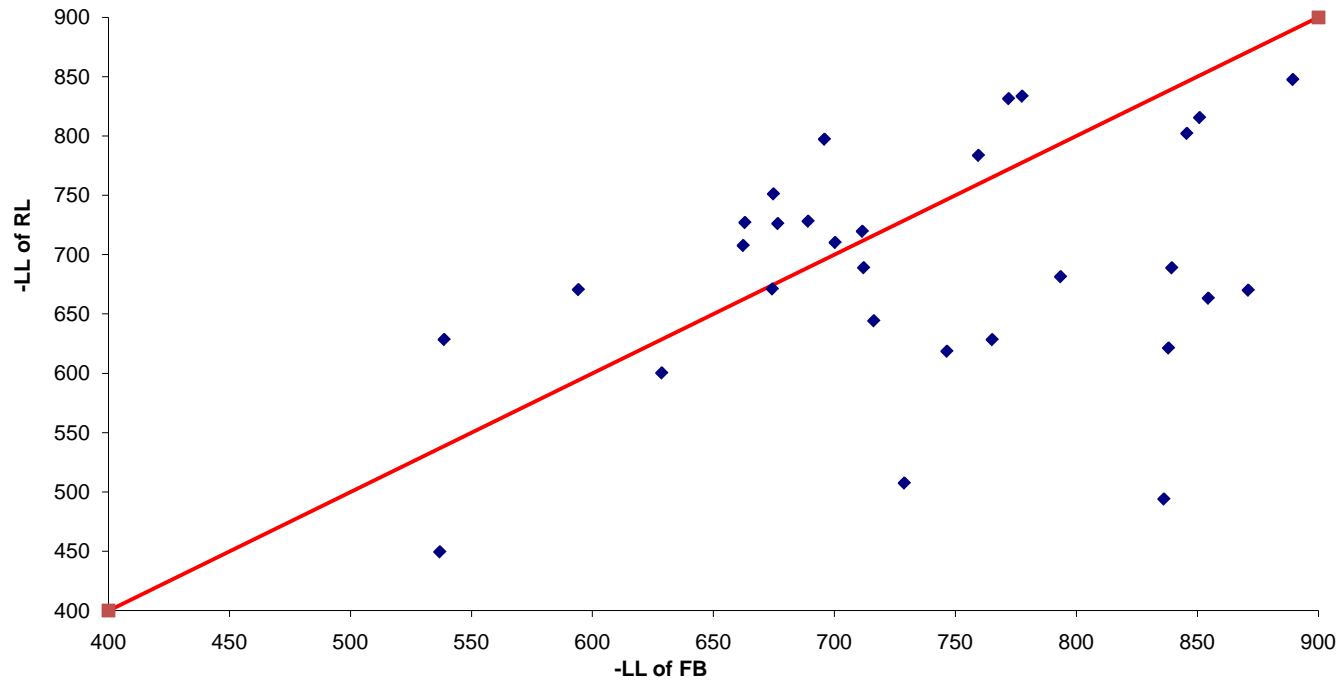

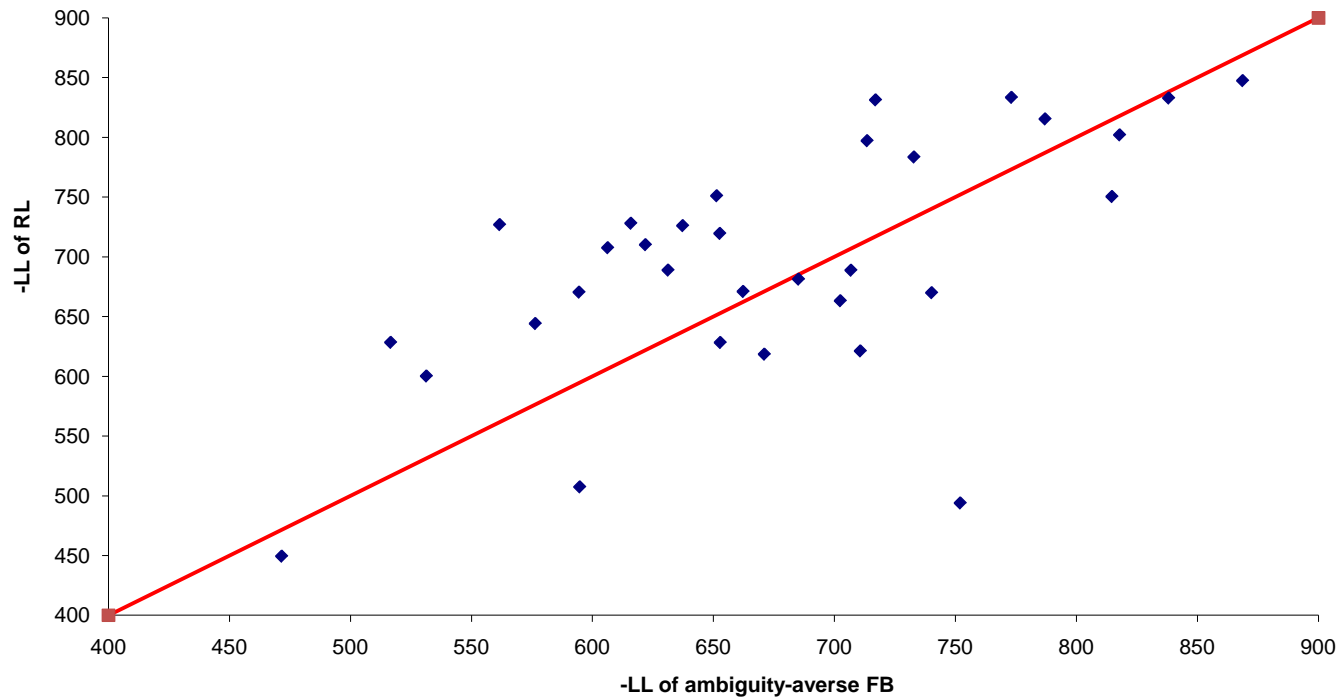

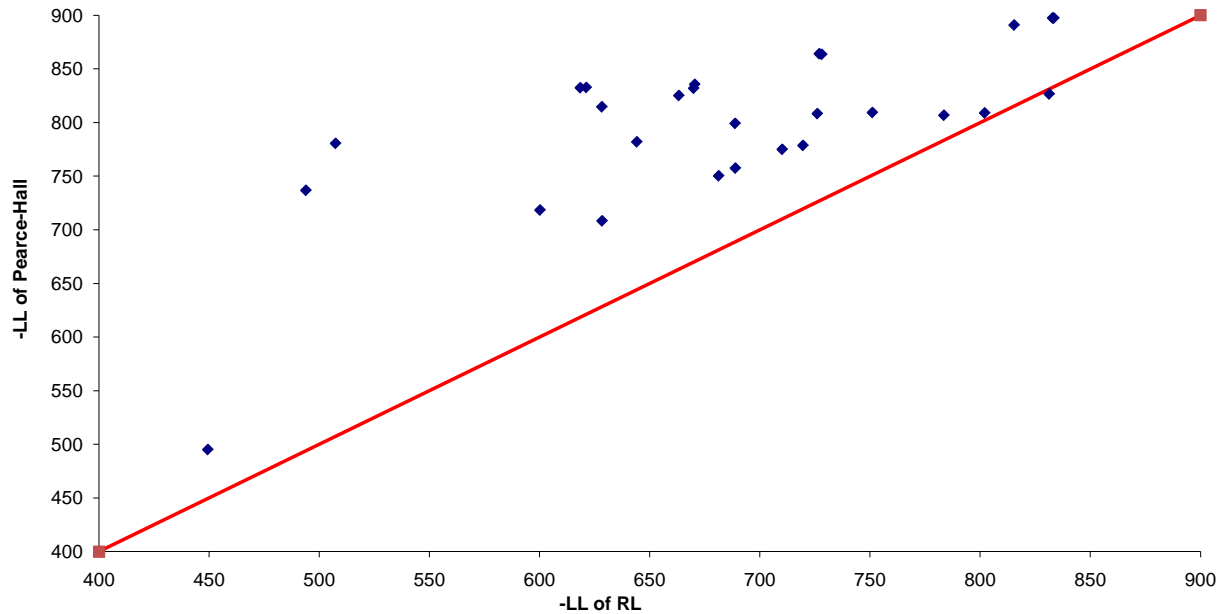

Supplement: Figure S3 — Graphical display of the individual (negative) log-likelihoods of the models in Treatment 1. (0.02 MB PDF) [file pcbi.1001048.s003.pdf]
