# Supplementary material for: Risk, Unexpected Uncertainty, and Estimation Uncertainty: Bayesian Learning in Unstable Settings
Source: PLoS Comput Biol. 2011 Jan 20;7(1):e1001048. doi: 10.1371/journal.pcbi.1001048 (PMC3024253; doi:10.1371/journal.pcbi.1001048)
Supplement: Text S1 — Supplemental material. (0.20 MB PDF) [file pcbi.1001048.s005.pdf]

Supplemental Material

Computation of Unexpected Uncertainty

To compute $\lambda(T)_{\mathrm{red}} = P(J_{\mathrm{red}\,T} = 0 \,|\, \underline{\mathbf{c}}_{\mathbf{lT}})$, let $P_{l\,T}(\mathbf{p})(J_{\mathrm{red}\,T} = 0)$ and $P_{l\,T}(\mathbf{p})(J_{\mathrm{red}\,T} = 1)$ denote the posterior probability distribution after *no jump* and after *jump*, respectively. Absent further information, the Bayesian model sets the prior belief of a jump equal to 0.5. Thus, the subjective jump likelihood at time T, $P(J_{\mathrm{red}\,T} = 0 \,|\, \underline{\mathbf{c}}_{\mathbf{lT}})$, equals to

$$\frac{1/2 \displaystyle\int_{\Theta} l(\mathbf{c}_{\mathbf{lT}}|\mathbf{p}) P_{l\,T}(\mathbf{p})(J_{\mathrm{red}\,T} = 0)\, d\mathbf{p}}{1/2 \displaystyle\int_{\Theta} l(\mathbf{c}_{\mathbf{lT}}|\mathbf{p}) P_{l\,T}(\mathbf{p})(J_{\mathrm{red}T} = 0)\, d\mathbf{p} + 1/2 \displaystyle\int_{\Theta} l(\mathbf{c}_{\mathbf{lT}}|\mathbf{p}) P_{l\,T}(\mathbf{p})(J_{\mathrm{red}\,T} = 1)\, d\mathbf{p}.}$$

We can rewrite the previous form as

$$\frac{1}{1 + \dfrac{\displaystyle\int_{\Theta} l(\mathbf{c}_{\mathbf{lT}}|\mathbf{p}) P_{l\,T}(\mathbf{p}_{\mathbf{lT}})(J_{\mathrm{red}\,T} = 1)\, d\mathbf{p}}{\displaystyle\int_{\Theta} l(\mathbf{c}_{\mathbf{lT}}|\mathbf{p}) P_{l\,T}(\mathbf{p})(J_{\mathrm{red}\,T} = 0)\, d\mathbf{p}}},$$

where

$$\begin{aligned} P_{l\,T}(\mathbf{p})(J_{\mathrm{red}T} = 1) &= P_0(\mathbf{p}), \\ P_{l\,T}(\mathbf{p})(J_{\mathrm{red}T} = 0) &= P_{l\,T/T}(\mathbf{p}) \;\; \left(= \frac{l(\mathbf{c}_{\mathbf{lT}}|\mathbf{p}) P_{l\,T-1}(\mathbf{p})}{\int_{\Theta} l(\mathbf{c}_{\mathbf{lT}}|\mathbf{p}) P_{l\,T-1}(\mathbf{p})\, d\mathbf{p}}\right). \end{aligned}$$

Therefore, we have:

$$\lambda_{\mathrm{red}}(T) = \frac{1}{1 + \dfrac{AC}{B}},$$

where

$$\begin{cases} A = \displaystyle\int_{\Theta} l(\mathbf{c}_{\mathbf{lT}}|\mathbf{p}) P_0(\mathbf{p})\, d\mathbf{p}, \\ B = \displaystyle\int_{\Theta} l(\mathbf{c}_{\mathbf{lT}}|\mathbf{p})^2 P_{l\,T-1}(\mathbf{p})\, d\mathbf{p}, \\ C = \displaystyle\int_{\Theta} l(\mathbf{c}_{\mathbf{lT}}|\mathbf{p}) P_{l\,T-1}(\mathbf{p})\, d\mathbf{p}. \end{cases}$$

Since $l(\mathbf{c_{lT}}|\mathbf{p}) = \prod_{i=1}^{3} p_i^{c_{liT}}$,, we write A as follows:

$$A = \int_{\Theta} \prod_{i=1}^{3} p_i^{c_{liT}} \frac{\prod_{i=1}^{3} p_{liT}^{\nu_0 \hat{p}_{i0}-1}}{\frac{\prod_{i=1}^{3} \Gamma(\nu_0 \hat{p}_{i0})}{\Gamma(\nu_0)}} d\mathbf{p}.$$

Because

$$\int_{\Theta} \prod_{i=1}^{3} p_i^{c_{liT}+\nu_0 \hat{p}_{i0}-1} \, d\mathbf{p} = \frac{\prod_{i=1}^{3} \Gamma(c_{liT} + \nu_0 \hat{p}_{i0})}{\Gamma(\sum_{i=1}^{3} c_{liT} + \nu_0)},$$

we can rewrite A as follows:

$$A = \frac{\Gamma(\nu_0) \prod_{i=1}^{3} \Gamma(c_{liT} + \nu_0 \hat{p}_{i0})}{\Gamma(\underbrace{\sum_{i=1}^{3} c_{liT}}_{1} + \nu_0) \prod_{i=1}^{3} \Gamma(\nu_0 \hat{p}_{i0})}.$$

Let i^* refer to the realized component of the count vector at time $T-1$. (For example, suppose that location l delivered the loss outcome at trial $T-1$; then $c_{lT-1} = (1,0,0)$, and i^* is equal to 1.) Because $\Gamma(x+1) = x\Gamma(x)$, and since $c_{liT} = 0, \ \forall i \neq i^*$ while $c_{li^*T} = 1$, we can further simplify this expression:

$$A = \frac{\Gamma(\nu_0) \prod_{i=1}^{3} \Gamma(c_{liT} + \nu_0 \hat{p}_{i0})}{\Gamma(1+\nu_0) \prod_{i=1}^{3} \Gamma(\nu_0 \hat{p}_{i0})} = \frac{\prod_{i=1}^{3} \Gamma(c_{liT} + \nu_0 \hat{p}_{i0})}{\nu_0 \prod_{i=1}^{3} \Gamma(\nu_0 \hat{p}_{i0})}.$$

Hence,

$$\begin{aligned} A &= \frac{\Gamma(1+\nu_0 \hat{p}_{i^*0}) \prod_{i \neq i^*} \Gamma(\nu_0 \hat{p}_{i0}) \Gamma(1+\nu_0 \hat{p}_{i^*0})}{\nu_0 \prod_{i=1}^{3} \Gamma(\nu_0 \hat{p}_{i0})} \\ &= \frac{1}{\nu_0} \frac{\Gamma(1+\nu_0 \hat{p}_{i^*0})}{\Gamma(\nu_0 \hat{p}_{i^*0})} \\ &= \hat{p}_{i^*0}. \end{aligned}$$

The calculation of B is analogous:

$$B = \frac{\Gamma(\nu_{lT-1}) \prod_{i=1}^{3} \Gamma(2c_{liT} + \nu_{lT-1}\hat{p}_{ilT-1})}{\Gamma(2 \underbrace{\sum_{i=1}^{3} c_{liT}}_{1} + \nu_{lT-1}) \prod_{i=1}^{3} \Gamma(\nu_{lT-1}\hat{p}_{ilT-1})}.$$

Since

$$\Gamma(2+\nu_{lT-1}) = \Gamma(1+(1+\nu_{lT-1})) = (1+\nu_{lT-1})\Gamma(1+\nu_{lT-1}) = (1+\nu_{lT-1})\nu_{lT-1}\Gamma(\nu_{lT-1}),$$

and

$$\prod_{i=1}^{3} \Gamma(2c_{liT} + \nu_{lT-1}p_{il\hat{T}-1}) = \prod_{i \neq i^*} \Gamma(\nu_{lT-1}\hat{p}_{ilT-1})(1 + \nu_{lT-1}\hat{p}_{i^*lT-1})\Gamma(1 + \nu_{lT-1}\hat{p}_{i^*lT-1}),$$

B simplifies to:

$$B = \frac{(1 + \nu_{lT-1}\hat{p}_{i^*lT-1})\hat{p}_{i^*lT-1}}{1 + \nu_{lT-1}}.$$

Using analogous arguments,

$$C = \frac{\Gamma(\nu_{lT-1}) \prod_{i=1}^{3} \Gamma(c_{liT} + \nu_{lT-1}\hat{p}_{ilT-1})}{\Gamma(\underbrace{\sum_{i=1}^{3} c_{liT}}_{1} + \nu_{lT-1}) \prod_{i=1}^{3} \Gamma(\nu_{lT-1}\hat{p}_{ilT-1})} = \hat{p}_{i^*\,lT-1}.$$

Consequently,

$$\frac{AC}{B} = \frac{\hat{p}_{i^*0}(\nu_{lT-1} + 1)}{1 + \nu_{lT-1}\hat{p}_{i^*lT-1}}.$$

Thus, $\lambda(T)$ depends on $\frac{\hat{p}_{i^*0}}{\hat{p}_{i^*lT-1}}$, the *odds ratio* (i.e., *strength of evidence*) for the hypothesis that a jump has occurred at time T.
